# Efficient simulation of a low-profile visualized intraluminal support device: a novel fast virtual stenting technique

**DOI:** 10.1186/s41016-018-0112-0

**Published:** 2018-03-22

**Authors:** Qianqian Zhang, Jian Liu, Yisen Zhang, Ying Zhang, Zhongbin Tian, Wenqiang Li, Junfan Chen, Xiao Mo, Yunhan Cai, Nikhil Paliwal, Hui Meng, Yang Wang, Shengzhang Wang, Xinjian Yang

**Affiliations:** 1 Department of Interventional Neuroradiology, Beijing Neurosurgical Institute and Beijing Tiantan Hospital, Capital Medical University, Beijing, China; 2 Capital Medical University School of Biomedical Engineering, Beijing Key Laboratory of Fundamental Research on Biomechanics in Clinical Application, Beijing, China; 3 Toshiba Stroke and Vascular Research Center, University at Buffalo, The State University of New York, Buffalo, New York USA; 4 Department of Mechanical and Aerospace Engineering, University at Buffalo, The State University of New York, Buffalo, New York USA; 5 Department of Neurosurgery, University at Buffalo, The State University of New York, Buffalo, New York USA; 6 Department of Neurosurgery, The First Affiliated Hospital, Nanchang University, Nanchang, China; 7 Institute of Biomechanics, Department of Aeronautics and Astronautics, Fudan University, Shanghai, China

**Keywords:** Intracranial aneurysm, LVIS, Hemodynamics, Endovascular treatment

## Abstract

**Background:**

The low-profile visualized intraluminal support (LVIS) stent has become a promising endovascular option for treating intracranial aneurysms. To achieve better treatment of aneurysms using LVIS, we developed a fast virtual stenting technique for use with LVIS (F-LVIS) to evaluate hemodynamic changes in the aneurysm and validate its reliability.

**Methods:**

A patient-specific aneurysm was selected for making comparisons between the real LVIS (R-LVIS) and the F-LVIS. To perform R-LVIS stenting, a hollow phantom based on a patient-specific aneurysm was fabricated using a three-dimensional printer. An R-LVIS was released in the phantom according to standard procedure. F-LVIS was then applied successfully in this aneurysm model. The computational fluid dynamics (CFD) values were calculated for both the F-LVIS and R-LVIS models. Qualitative and quantitative comparisons of the two models focused on hemodynamic parameters.

**Results:**

The hemodynamic characteristics for R-LVIS and F-LVIS were well matched. Representative contours of velocities and wall shear stress (WSS) were consistently similar in both distribution and magnitude. The velocity vectors also showed high similarity, although the R-LVIS model showed faster and more fluid streams entering the aneurysm. Variation tendencies of the velocity in the aneurysm and the WSS on the aneurysm wall were also similar in the two models, with no statistically significant differences in either velocity or WSS.

**Conclusions:**

The results of the computational hemodynamics indicate that F-LVIS is suitable for evaluating hemodynamic factors. This novel F-LVIS is considered efficient, practical, and effective.

## Background

Intracranial aneurysms (IAs), a common disorder with a general prevalence ranging from 0.5% to 6.9%, are abnormal dilatations caused by pathological weakness and disruption of a vessel’s wall [1, 2]. When an IA is diagnosed, the patients must be protected from intracranial hemorrhage, which is associated with a fatality rate as high as 67% [1]. Treatment aimed at excluding an aneurysm from the cerebral circulation can be performed before hemorrhage occurs. Among these techniques, endovascular treatment is widely accepted because of its minimal invasiveness and safety [3]. A low-profile visualized intraluminal support (LVIS) device—a self-expanding nickel–titanium (nitinol) stent—initially designed to provide a scaffold of coils, has emerged as a promising endovascular option for treating IAs [4]. Braided LVIS devices is more compliant and flexible than other micro-stents (e.g., Enterprise, a laser-cut stent). This stent, with four radiopaque tantalum markers, also offers better visualization [5].

Although extensively utilized, treatment with LVIS is not free from risk. Complications may occur during and after insertion, notably bleeding, recanalization, and ischemia. There have been few direct, accurate assessments of alterations in aneurysm hemodynamics caused by an LVIS device, largely because of the limits of current imaging techniques [6, 7]. Meanwhile, flow hemodynamics have been shown to play important roles in the aneurysm’s history. Thus, having an in-depth understanding of hemodynamic changes induced by LVIS necessitates the development of simulation tools that could help guide clinicians toward strategies which would ensure aneurysm obliteration and minimize complications. Image-based computational fluid dynamics (CFD) modeling provides a potential tool for reproducing flow hemodynamics and has been reported to be trustworthy for evaluating hemodynamic changes in vitro [8–10]. To date, however, there has been no validated effective and efficient numerical simulation for LVIS.

In this study, we aimed to develop a novel fast virtual stenting technique (FVST) to simulate LVIS. We sought to determine the reliability of a representation of the real stent that would accurately reflect the hemodynamic status of the aneurysm, thereby helping the clinician make judicious treatment decisions. Based on the advantages of CFD, the objective of this research was to explore the practicability and effectiveness of the state-of-the-art FVST with LVIS (F-LVIS).

## Methods

**Fig. 1:**
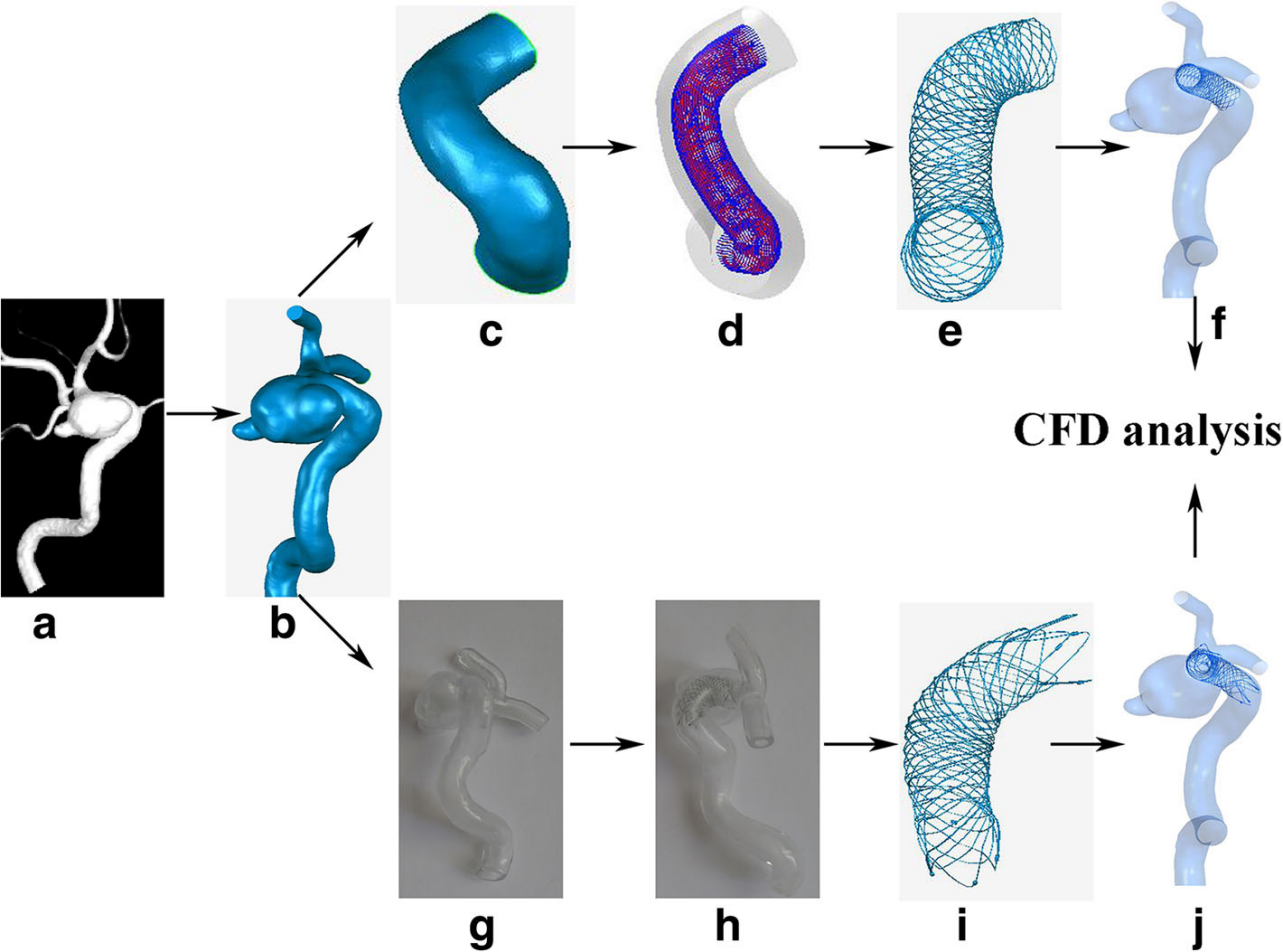
Work flow of the study. **a**: Digital images of the patient-specific aneurysm acquired from three-dimensional (3D) rotational angiography; **b**: 3D reconstruction of the aneurysm saved as stereolithography format file; **c**: Parent vessel isolated from the aneurysm’s geometry; **d**: Simplex mesh initiation and expansion; **e**: Fast virtual stenting technique with LVIS; **f**: Modeled LVIS merged with the patient’s vascular system; **g**: patient-specific hollow phantom fabricated using a 3D printer; **h**: patient-specific hollow phantom deployed with real LVIS stent; **i**: 3D reconstruction of the real LVIS scanned by micro-CT; **j**: Real LVIS merged with the patient’s vascular system

The flow of our study is shown in Fig. 1.

### Patient-specific aneurysm phantom

A patient-specific aneurysm that we had previously developed and used for validating an Enterprise stent via an FVST was selected for use in the present study [11]. Digital images (Fig. 1a) acquired from three-dimensional (3D) rotational angiography were collected to create 3D reconstruction of the aneurysm and then saved to a stereolithography format file (Fig. 1b). Some inessential small arteries which were reconstructed relatively difficult and inaccurate, such as ophthalmic artery and posterior communicating artery, were removed to ensure the computational efficiency of CFD, following which a patient-specific hollow phantom was fabricated using a 3D printer (Materialise, Lueven, Belgium) (Fig. 1g). The aneurysm was wide-necked (width 12.27 mm, height 9.35 mm) and located at the C6 segment of the internal carotid artery. Of note, this phantom model is consistent with the vascular anatomy of the patient, not only regarding aneurysmal morphology but also vascular curvature.

### Deployment of stent: Real and simulated LVIS respectively

After fabrication of the specific model, a 4.5 × 23 mm LVIS stent, whose dimensions were dictated by the vascular vessel and aneurysm size, was ready for endovascular placement. The stent was released successfully by an experienced neurosurgeon and his assistant according to operative standards (Fig. 1h). The phantom model deployed with LVIS was then scanned by a micro cone-beam computed tomography (micro-CT) system with high resolution (8.8 μm). The implanted stent was reconstructed manually to generate a real LVIS (R-LVIS) stereolithography file using Mimics 10.01 (Materialise, Leuven, Belgium) (Fig. 1i).

The F-LVIS constructed is similar to that of the Enterprise stent that we previously reported [11, 12]. The flow of work to construct the F-LVIS is based on the concept of simplex meshes generating a deformable structure that is finally swept into 3D stent wires. The work flow occurs mainly in three steps.

1. *Preprocessing*: The aneurysm’s geometry is isolated from the parent artery using Geomagic Studio (Raindrop Geomagic, Research Triangle Park, NC, USA). Subsequently, a centerline suitable to the parent artery is extracted using Mimics 10.01 (Fig. 1c).
2. *Simplex mesh initiation and expansion*: Simplex mesh representing the surface of LVIS based on the centerline is initiated along the parent vessel. The simplex mesh then undergoes radial expansion via MATLAB 2013 (MathWorks, Natick, MA, USA). The expansion stops when the deformable mesh is in optimal apposition with the vessel wall (Fig. 1d).
3. *LVIS pattern mapping*: The expanded simplex mesh is then input into Abaqus/Explicit 6.12 (Simulia, Providence, RI, USA) to obtain wire curves using an in-house LVIS python code. Next, the wire curves are swept into 3D structures in the computer-aided designed program Creo Parametric 2.0 (PTC, Needham, MA, USA). Finally, the LVIS modeled by 16 overlaying wires is completed (Fig. 1e).

F-LVIS and R-LVIS were then merged with the patient’s vascular system to create computational models (Fig. 1f, g). CFD calculations were performed to analyze the hemodynamic variations.

### CFD calculation and hemodynamic analysis

We performed CFD simulations in the F-LVIS and R-LVIS models in a manner similar to those described previously [13, 14]. ICEM software (version 14.5; ANSYS Inc., Canonsburg, PA, USA) was used to create finite-volume tetrahedral elements. After meshing, the hemodynamics was simulated with ANSYS CFX software (ANSYS CFX 14.0; ANSYS, Inc.). The Navier–Stokes formulation was solved to simulate fluid flow, with blood assumed to be a homogenous, laminar, incompressible Newtonian fluid. A measured viscosity (4 cPa, specific density 1060 kg/m^3^) was applied under rigid-walled, no-slip boundary conditions. Zero pressure was set at the outlet, and a representative pulsatile period velocity profile obtained by transcranial Doppler imaging was implemented as the inflow boundary condition. To confirm the numerical stability, we performed two cardiac cycle simulations. The results of the final cardiac cycle was output to underlie hemodynamic analyses.

When CFD calculations were completed, the following meaningful parameters were collected using ANSYS CFD-post software (ANSYS CFX 14.0; ANSYS, Inc.): velocity streamlines, vectors of the flow velocity, wall shear stress (WSS). Qualitative and quantitative results of the hemodynamics were compared between F-LVIS and R-LVIS.

### Statistical analysis

Data analysis was performed with statistical software (SPSS V.19.0; IBM, Chicago, IL, USA). The one-sample Kolmogorov–Smirnov test was used to test normal-distribution data for continuous parameters, and the paired-samples t-test was used when the data were approximately normally distributed. Continuous variables were presented as medians (interquartile range). Two-tailed *p* < 0.05 was considered to indicate statistical significance.

## Results

The F-LVIS work flow took only about 6 min to generate the virtual LVIS, compared with approximately 6 h for the R-LVIS to perform micro-CT scanning and reconstruction. Hemodynamics parameters that focused on velocities and the WSS were extracted to detect discrepancies between the two models.

**Fig. 2:**
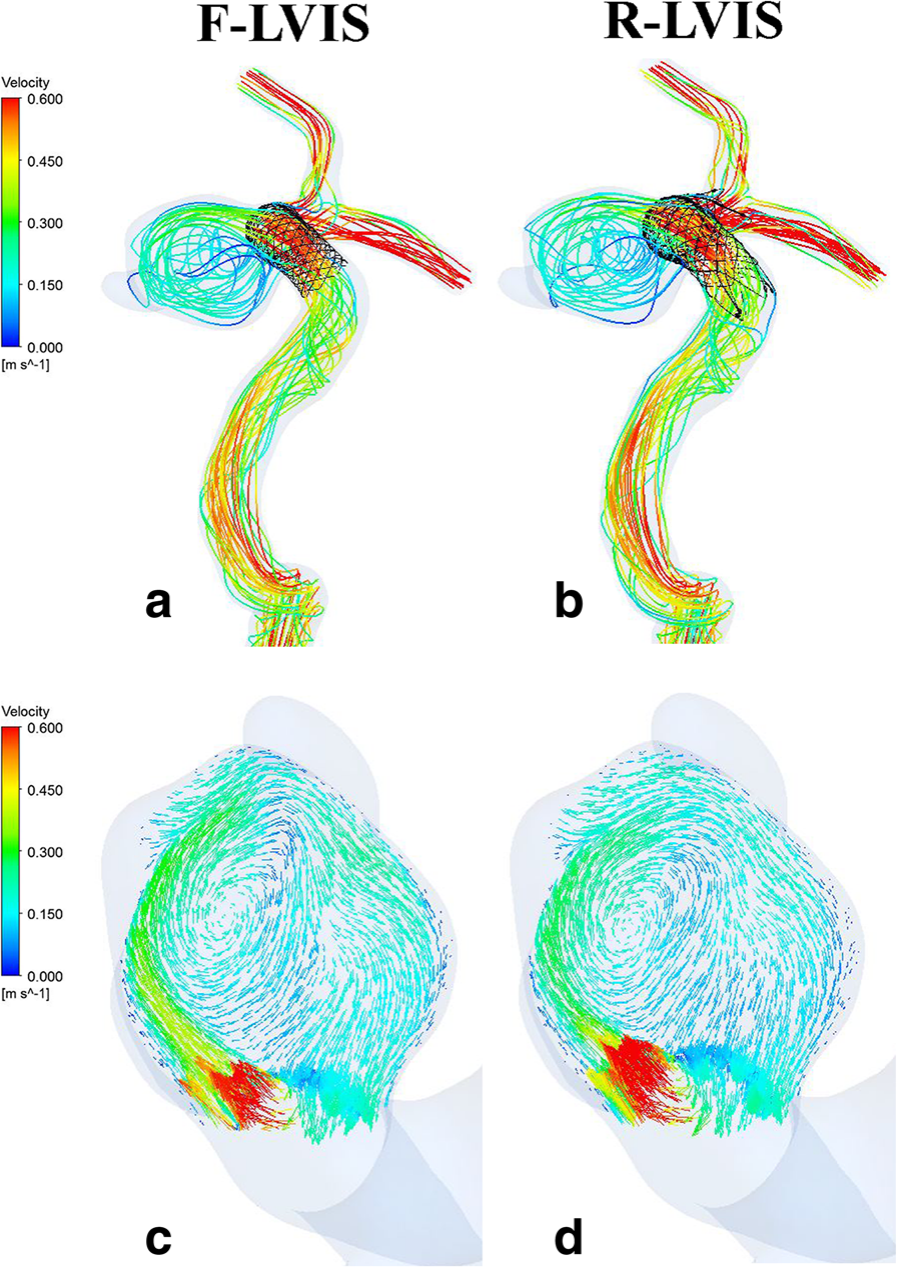
Representative contours of velocity streamlines and vectors at peak systole. *Top row*: Velocity streamlines after fast low-profile visualized intraluminal support (F-LVIS) deployment (**a**) and real low-profile visualized intraluminal support (R-LVIS) deployment (**b**). *Bottom row*: Velocity vectors in the cross section of the F-LVIS-released aneurysm (**c**) and the R-LVIS-released aneurysm (**d**)

**Fig. 3:**
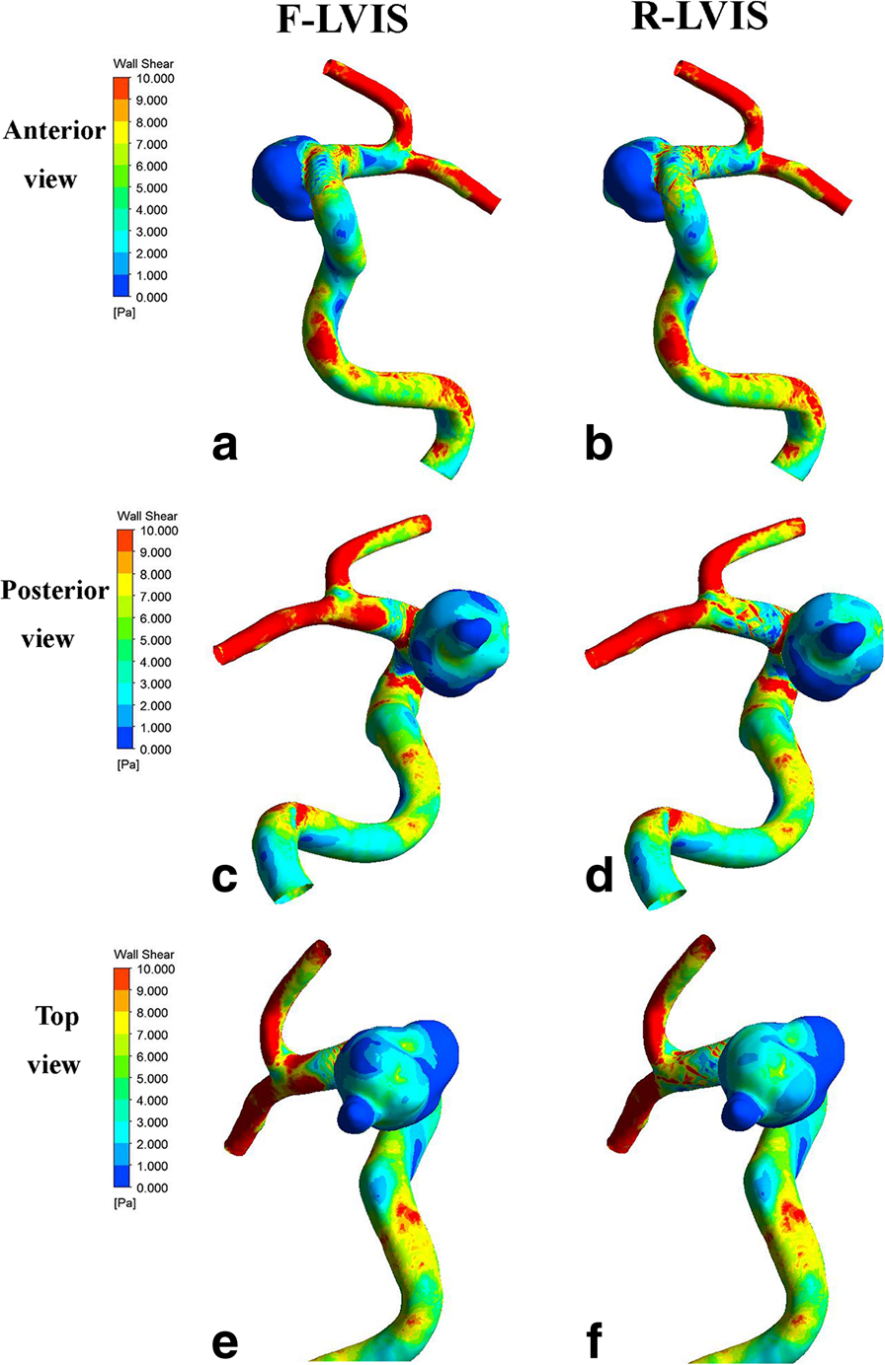
Wall shear stress from the anterior (**a**, **b**), posterior (**c**, **d**), and top (**e**, **f**) views of the aneurysm. *Top row*: Anterior view of the F-LVIS (**a**) and R-LVIS (**b**) models. *Middle row*: Posterior views of F-LVIS (**c**) and R-LVIS (**d**). *Bottom row*: Top views of F-LVIS (**e**) and R-LVIS (**f**)

Hemodynamic results, with representative contours of the velocities and WSS at peak systole, are shown, respectively, in Fig. 2 and Fig. 3. Overall, the instantaneous velocity streamlines were similar in the two models. The higher velocities correspond to the parent vessel in the F-LVIS model, created possibly by deployment of the stent, whereas lower-magnitude velocities are observed in the aneurysm after blood passes through the LVIS pores (Fig. 2a, b). The velocity vectors in the cross sections perpendicular to the aneurysmal necks matched well in the two cases (Fig. 2c, d). In general, clockwise and single-vortex flow patterns were clearly detected despite different approaches to deploying the stent. The flow entered along the aneurysm wall with high speed, spread, and exited from the neck at a relative slow speed. Only small differences could be detected, although faster, more fluid streams entered the aneurysm in the R-LVIS model compared with those in the F-LVIS model.

Like the velocity, WSS depicted on the aneurysm and parent vessel were similar in the two models (Fig. 3). Regions of low or high WSS, especially visualized from the anterior and posterior views, were matched perfectly as to locations and area sizes (Fig. 3a–d). On the top view, there was a slight difference in WSS distribution, showing larger low-WSS areas in the aneurysm dome.

**Fig. 4:**
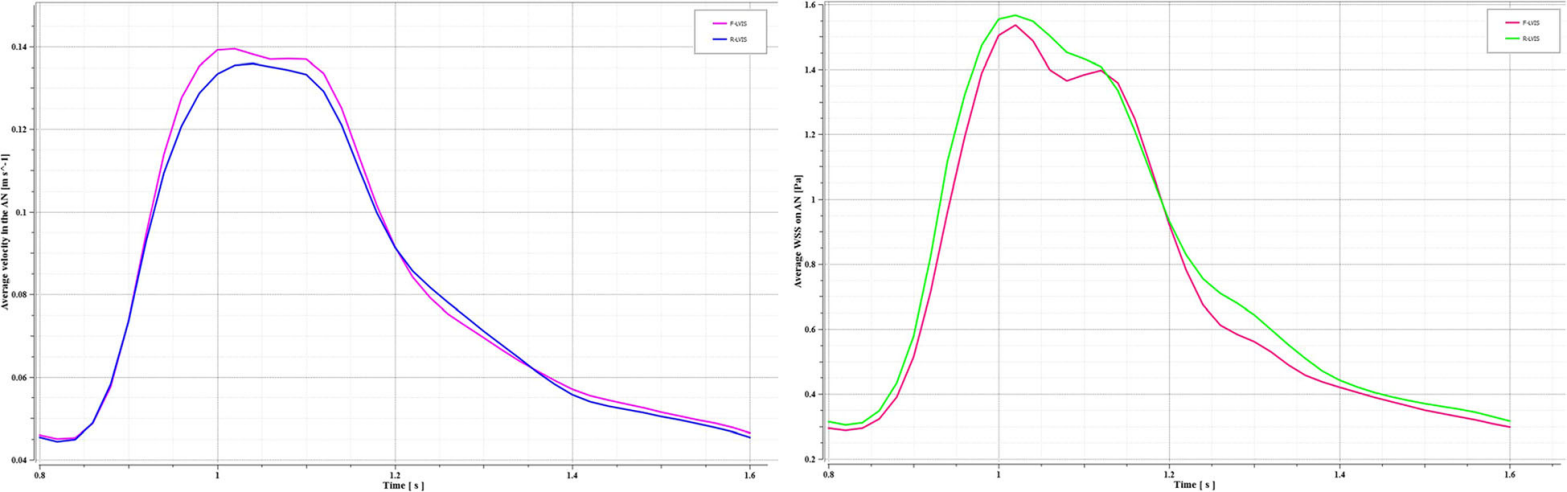
Representative curves of velocity in the aneurysm and wall shear stress (WSS) on the neck wall within a single cardiac cycle. Left: velocity curves in F-LVIS and R-LVIS models. Right: WSS curves in F-LVIS and R-LVIS models

Quantitative comparisons between the F-LVIS and R-LVIS results were accomplished by plotting average velocity curves inside the aneurysm and WSS curves on the whole aneurysm within a single cardiac cycle (Fig. 4). The velocity curves showed almost the same mean velocity trends, providing evidence that the F-LIVS simulations were in conformance with the R-LVIS outcomes. In line with the findings of average velocity, WSS curve quantities showed good agreement, except for a slightly larger fluctuation during the periods of 1.05–1.1 s and 1.25–1.3 s, which was underestimated in the F-LVIS models.

For further statistical analysis, we extracted maximum and minimum values of the velocity in the aneurysm and the WSS around the aneurysm neck from the cardiac cycle (Table 1). The F-LVIS results showed that the median maximum velocity reached 0.14 m/s, and the median minimum velocity was 1.44 × 10^− 5^ m/s. In contrast, the R-LVIS results showed values of 0.12 m/s and 9.72 × 10^− 6^ m/s, respectively. The median value for the maximum WSS was slightly larger for the F-LVIS than the R-LVIS (12.35 vs 12.10 pa), whereas the median minimum WSS was smaller for the F-LVIS than for the R-LVIS (2.69 × 10^− 3^ vs 3.14 × 10^− 3^ pa). There were no significant differences for any of other comparisons between F-LVIS and R-LVIS (*p* > 0.05) (Table 1).

**Table 1:** Velocities in the aneurysm and wall shear stress around the aneurysm neck for the F-LVIS and R-LVIS models

Variables	F-LVIS	R-LVIS	*p* Value
Velocity, m/s			
Maximum	0.14(0.09–0.26)	0.12(0.08–0.26)	0.42
Minimum	1.44 × 10^−5^(4.54 × 10^−6^-2.86 × 10^− 5^)	9.72 × 10^− 6^(3.68 × 10^− 6^-2.95 × 10–5)	0.42
WSS, pa			
Maximum	12.35(9.54–26.46)	12.10(8.60–23.35)	0.06
Minimum	2.69 × 10^−3^(1.04 × 10^− 3^-1.17 × 10^−2^)	3.14 × 10^− 3^(9.48 × 10^−4^-4.87 × 10^− 3^)	0.25

F-LVIS: fast virtual stenting technique with LVIS; R-LVIS: real low-profile visualized intraluminal support; WSS: wall shear stress

## Discussion

Several virtual stenting techniques have been developed to help clinicians with decision-making and endovascular treatment plans, ranging from the use of various stent types (e.g., Enterprise, Neuroform) to a flow diverter [12, 15, 16]. In this study, we developed a novel fast virtual stenting technique especially designed for LVIS and validated its effectiveness. The novel F-LVIS simulation allows a precise description of the aneurysm’s hemodynamics, consistently quantifying the performance of a real commercial LVIS device. Overall, this virtual stenting technique is desirable because of its high efficiency, practicality, and effectiveness. To our knowledge, this is a unique report of validating LVIS simulation methodology.

The mechanics of virtual stent release can be mathematically defined by means of differential equations [15, 17]. In an optimal situation, a tool for virtual stenting should be efficient enough to allow clinicians to compare various treatment strategies and choose the best one for the patient without delay. It is known that finite-element methods are used extensively to simulate stents, but these methods are too time consuming to be user-friendly for clinicians [16]. With respect to the time cost of virtual stenting, we applied adaptive expansion to generate LVIS wires, which drastically reduced the computational time of the stenting procedure by decreasing the number of expansion steps. As a result, the time it takes to produce the LVIS is acceptable and usable by clinicians attempting to choose the most effective treatment.

Another advantage of the F-LVIS is its practicality. Over the years, a number of stent modeling techniques have been proposed that were based on an ideal aneurysm model [18–20]. According to previous studies, vascular and aneurysm geometries are regarded as strong factors that characterize intra-aneurysmal hemodynamics, which are closely correlated with the aneurysm’s natural history [21, 22]. F-LVIS exhibited a benefit in fitting well with the curved vasculature, thereby boosting our confidence in the simulation. Different from Augsburger et al., who modeled the stent as a porous medium [23], our novel technique could produce a configuration similar to that of the LVIS, which could also be applied successfully to a patient-specific aneurysm, ensuring the potential application to a diversity of aneurysm shapes and complicated vascular structures.

Finally, it is noteworthy that the hemodynamics results from the F-LVIS showed evident agreement with the real LVIS device, both quantitatively and qualitatively. It is well known that flow hemodynamics variations caused by the presence of a stent play a role in the occlusion of the aneurysm as they are involved with bridging the aneurysm neck, progressive flow stasis, and thrombus formation [24]. It has been shown that, independent of the deploying approach, there are no significant differences in the velocity and WSS parameters between F-LVIS an R-LVIS, suggesting the accuracy of the currently adopted method. Also exciting are the simplifications during the whole stent deployment procedure that reduce the computational time but maintain consistency in the hemodynamics variations. We believe this novel technique will be a promising tool for endovascular neurosurgeons to make optimal decision before aneurysm treatment, and predict hemodynamic changes which is helpful for the evaluation of treatment outcomes after deploying LVIS.

### Limitations

This study has some potential limitations. First, we develop a novel fast virtual stenting technique (FVST) to simulate LVIS and validate its reliability in a patient-specific model. Though the hemodynamic characteristics between R-LVIS and F-LVIS matched well, only one aneurysm phantom model was validated. However, we consider the results credible because of the common site (internal carotid artery) the aneurysm located in and the widely use of LVIS in the wide-necked aneurysm. In addition, we used simplified CFD analyses like other CFD simulation studies, including some eliminating inessential small vascular structures, the neglection of the shorten after LVIS deployment, rigid walls, Newtonian blood properties, and physiological flow-boundary conditions. The method and results of our research remain to be further verified and applied in future.

## Conclusion

The F-LVIS presented in this research may be a good compromise as a feasible model for the stenting procedure, with no loss of detailed hemodynamic features. The study reinforces the notion that numerical simulation could be a promising tool to help with treatment planning in the clinical setting.

## Abbreviations

3D: three-dimensional
CFD: computational fluid dynamics
F-LVIS: fast virtual stenting technique with LVIS
FVST: fast virtual stenting technique
IA: intracranial aneurysm
LVIS: low-profile visualized intraluminal support
micro-CT: micro cone-beam computed tomography
R-LVIS: real low-profile visualized intraluminal support
WSS: wall shear stress
